# Transparent Exopolymer Particles in Deep Oceans: Synthesis and Future Challenges

**DOI:** 10.3390/gels7030075

**Published:** 2021-06-22

**Authors:** Toshi Nagata, Yosuke Yamada, Hideki Fukuda

**Affiliations:** 1 Atmosphere and Ocean Research Institute, The University of Tokyo, Kashiwa 277-8564, Japan; 2 Marine Biophysics Unit, Okinawa Institute of Science and Technology Graduate University, Onna-son 904-0495, Japan; yosuke.yamada@oist.jp; 3 International Coastal Research Center, Atmosphere and Ocean Research Institute, The University of Tokyo, Otsuchi 028-1102, Japan; hfukuda@aori.u-tokyo.ac.jp

**Keywords:** transparent exopolymer particles, ocean carbon cycles, deep oceans

## Abstract

Transparent exopolymer particles (TEP) are a class of abundant gel-like particles that are omnipresent in seawater. While versatile roles of TEP in the regulation of carbon cycles have been studied extensively over the past three decades, investigators have only recently begun to find intriguing features of TEP distribution and processes in deep waters. The emergence of new research reflects the growing attention to ecological and biogeochemical processes in deep oceans, where large quantities of organic carbon are stored and processed. Here, we review recent research concerning the role of TEP in deep oceans. We discuss: (1) critical features in TEP distribution patterns, (2) TEP sources and sinks, and (3) contributions of TEP to the organic carbon inventory. We conclude that gaining a better understanding of TEP-mediated carbon cycling requires the effective application of gel theory and particle coagulation models for deep water settings. To achieve this goal, we need a better recognition and determination of the quantities, turnover, transport, chemical properties, and microbial processing of TEP.

## 1. Introduction

Seawater carries a wide variety of dissolved and particulate organic carbon (DOC and POC, respectively), covering a size range of less than a nanometer (dissolved organic molecules and colloids) to meters (whales), with a broad range of turnover times from minutes to millennia [1,2]. Understanding the magnitude and the spatiotemporal patterns of the production, transformation, and remineralization of organic matter is important not only for studies examining ocean carbon cycles but also for those investigating ocean ecosystems and Earth’s climate. One emerging concept in this research field is that gel-like particles play an important role in the regulation of organic carbon dynamics in the oceans. Gel-like particles are omnipresent in marine environments, being produced by microbes and larger organisms, released from decayed cells and tissues, or formed through the spontaneous self-assembly of DOC and subsequent coagulation of small particles [2,3,4]. Transparent exopolymer particles (TEP), consisting of acidic polysaccharides, are among the most abundant class of gel-like particles in marine environments [5,6]. TEP are mainly produced by phytoplankton and bacteria in the oceans [6]. Due to their sticky nature, TEP facilitate particle coagulation and may enhance the vertical transport of organic carbon and the carbon sequestration in the ocean [7]. TEP are porous and less dense, allowing them to accumulate in the sea surface microlayer, where they influence the air–sea exchange of climate-related gas [5]. TEP may also be a key component of marine food webs, providing habitats and organic substrates for microbes [8,9] and serving as a food for metazoans [10].

Although these processes and dynamics of TEP in marine systems have been extensively studied during the past three decades (reviewed by Passow et al. [6] and Mari et al. [5]), investigators have only recently begun to recognize intriguing features of TEP distribution and processes involved in the regulation of TEP dynamics in deep oceans. The emergence of new research reflects the growing attention to ecological and biogeochemical processes in deep oceans, where large quantities of organic carbon are stored and processed, yet mechanisms underlying these processes are not entirely clear (see reviews [11,12,13,14]). Here, we review recent research on TEP in deep ocean realms. We first provide an overview of recent TEP distribution data, which is followed by a discussion concerning sources and sinks of TEP. We then examine the contribution of TEP to the organic carbon pool. Our goal is to identify major knowledge gaps and future research challenges.

## 2. Overview of the Data on TEP Distribution in Deep Waters

In this paper, the mesopelagic layer refers to the depth layers between 200 and 1000 m, and the bathypelagic layer between 1000 m and the abyssal seafloor (up to the depth of 5400 m including a part of the abyssopelagic layer), unless different boundary depths are used in the source literature.

### 2.1. Data Obtained by the Colorimetry

The routine method to determine TEP concentration in seawater is colorimetry [15]. Briefly, TEP are collected on 0.4-µm-pore-size polycarbonate filters by filtration and are stained with Alcian blue, a cationic dye that binds to anionic carboxyl or half ester-sulfate groups of acidic polysaccarhides at low pH. After a short period of staining, filters are rinsed with pure water and soaked in acid. The redissolved dye concentration is determined colorimetrically. The TEP concentration is expressed as an equivalent amount of the standard substance, xanthan gum (a polysaccharide excreted by a bacterium, *Xanthomonas campestris*), with units of μg Xeq. L^−1^. The method measures the amount of dye bound to particles, which is converted to the “mass” using the binding capacity of the standard substance. The mass of TEP determined in this manner may deviate from the “true mass”, depending on the anionic density of TEP in natural seawater [16]. Thus, it is important to keep in mind that the colorimetric method is semiquantitative.

#### 2.1.1. Data Collected in Coastal, Slope Region, and Marginal Seas

An early study conducted by Passow and Alldredge [15] examined the depth profile of TEP in Santa Barbara Chanell in the eastern North Pacific, in summer. Their data revealed a low TEP concentration (ca. 20 μg Xeq. L^−1^), with little variation in concentration between the depths of 200 m and 1400 m. Substantially higher TEP concentrations in the mesopelagic layer (300 and 1000 m) were found by Bar-Zeev et al. [17] during transect cruises conducted in the oligotrophic eastern Mediterranean Sea. They found that TEP concentrations at seven sampling stations were, on average, 200 μg Xeq. L^−1^ at depths of both 300 and 1000 m. A notable feature was that the TEP concentration in the mesopelagic layer tended to decrease with increasing distance from the shore. This off-shoreward decreasing trend in TEP concentration was also observed in the near-surface layer.

Ortega-Retuerta et al. [18] examined the TEP distribution at 29 stations along the east–west transect across the Mediterranean Sea and the adjacent North Atlantic. They found that the TEP concentrations in the meso- and bathypelagic layers were 1.2–35 and 0.6–16 μg Xeq. L^−1^, respectively. The TEP concentration tended to decrease with depth, with a slope variation among the stations. The depth-integrated TEP values in the epipelagic and the meso- and bathypelagic waters were significantly positively correlated. Based on these results, the authors suggested that the TEP concentrations at depth are likely to be controlled by the vertical delivery of TEP.

Yamada et al. [19] investigated TEP concentrations at eight sampling stations in the slope region of the western Arctic Ocean. They found that the TEP concentration range in the layer between 200 and 1960 m was 37–129 μg Xeq. L^−1^, which displayed a decreasing tendency with depth. They suggested that the large amount of TEP produced in the Chukchi shelf are laterally transported to the slope region [20], where they then transfer to deeper layers due to the vertical transport of TEP associated with sinking particles and the subsequent dissociation from sinking particles during their transit through deep water columns.

In an estuarine environment (the lower St. Lawrence Estuary in Canada) with a maximum depth of 340 m, Annane et al. [21] examined the vertical distribution of TEP. They found a seasonal variation in the TEP concentration in the mesopelagic layer (130–320 m) over a range from 15 to 200 μg Xeq. L^−1^, with the highest concentration being observed in spring.

#### 2.1.2. Data Collected in Open Oceans

Cisternas-Novoa et al. [22] examined the full-depth distribution of TEP concentrations at the Bermuda Rise, the open ocean domain of the North Atlantic Ocean, where the maximum depth is about 4500 m. Seawater samples were collected on five occasions covering a seasonal cycle (February, May, August, and November). TEP concentrations below a depth of 200 m were uniformly low (mostly in the range of 20–30 μg Xeq. L^−1^) and seasonally less variable, with a notable exception found in February. In this month, they observed a TEP concentration peak at a depth of 2000 m. In May and June, a strong benthic nepheloid layer developed at depths below 4000 m. The benthic nepheloid layer is characterized by high turbidity and high particulate matter concentration near the seafloor, which is caused by particle erosion/resuspension and inhibited particle settling due to the bottom current and bottom boundary layer turbulent mixing [23]. The authors’ results showed that particle concentration and Coomassie stainable particle (CSP) concentration were indeed high in the benthic nepheloid layer, whereas such a trend was not evident for TEP concentrations.

**Figure 1:**
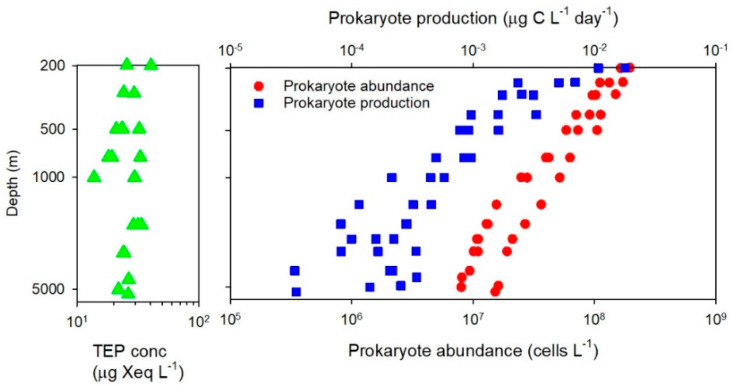
Vertical distributions of TEP concentrations, prokaryote abundance, and prokaryote production in the central Pacific Ocean. The graphs were made using the original data of Yamada et al. [19]. Both x and y axes are logarithmic.

At three sampling stations located in the subtropical and equatorial regions of the central Pacific, Yamada et al. [19] examined full depth distributions of TEP concentrations. Similar to the results obtained by Cisternas-Novoa et al. [22] in the Atlantic Ocean, the TEP concentration was uniform below a depth of 200 m down to the maximum depth of about 5400 m (range, 12–40 μg Xeq. L^−1^) (Figure 1). The vertical TEP distribution was largely decoupled from those of prokaryote abundance and production, which decreased by approximately 10-fold (abundance) or 100-fold (production) within the corresponding depth range (Figure 1).

### 2.2. Data Obtained by the Microscopy

The microscopic method relies on the binding of Alcian blue to TEP [8]. Seawater samples are filtered through 0.4- or 0.2-μm-pore-size polycarbonate filters, stained with Alcian blue, and observed under the light microscope. The size and abundance of TEP are determined either manually or with the aid of an image analysis system, after capturing TEP images with a camera. Because TEP images show the cross-sections of particles on a plane, the area of individual TEP can be determined to calculate the equivalent spherical diameter (ESD) and volume. Using ×200 magnification light microscopy and an image analysis system, the ESD range determined by the microscopy is typically between 1 μm and a few hundred μm, although the size range may vary depending on the study. The microscopic method is time-consuming, even with the aid of an image analysis system; only a few studies have used the microscopic method to analyze TEP in deep oceanic waters [17,24,25]. Although there has been an attempt to use an automated image acquisition system combined with a flow chamber (FlowCAM) to determine TEP abundance and size distribution in a rapid manner, this method has yet to be applied to the analysis of TEP in deep waters [22].

Engel et al. [25] reported the first comprehensive data of TEP abundance determined using a microscopic method in the meso- and bathypelagic oceans. They sampled at six vastly distant oceanic provinces, including subtropical/equatorial, coastal upwelling, and polar regions. Notably, an up to 40-fold higher TEP abundance (in terms of area per unit volume) was found in the Mauritanian upwelling region relative to other regions; moreover, this large difference held for both the meso- and bathypelagic layers. The TEP abundance in the mesopelagic layer was 1.3–3.3-fold higher than the TEP abundance in the bathypelagic layer in the regions examined, except for the subtropical/equatorial region of the Indian Ocean, where TEP abundance in the bathypelagic layer was about 2-fold higher than that in the mesopelagic layer.

Engel et al. [25] also examined the size distribution of TEP (size range, 1–500 μm). They found that the data did not fit well to the general power law model [26], irrespective of sampling depth. This was primarily because the size distribution slope within the size range of 1–6 μm was smaller than that within a larger size range. The relative abundance of large TEP tended to increase with TEP abundance. In the Mauritanian upwelling region where the TEP abundance was high, the size distribution of TEP was skewed toward larger size classes in both the meso- and bathypelagic layers.

In the Fram Strait in the Arctic Ocean, Busch et al. [24] examined the prokaryote colonization of TEP. They found that TEP was colonized by prokaryotes throughout the water column (the maximum depth, 2613 m). Interestingly, the highest density of prokaryotes attached to TEP (58 × 10^4^ cells mm^−2^) was found at a depth of 1000 m. Prokaryotes attached to TEP accounted for 1–20% (average, 3%) of the total prokaryote abundance. This value tended to increase with depth, with the highest value being found at depths >2000 m.

In the Mediterranean Sea, Bar-Zeev et al. [17] found that large TEP with amorphous shapes were abundant in the mesopelagic layer. The large TEP were often associated with prokaryotes, suggesting that TEP may serve as an organic carbon substrate for prokaryotes in the mesopelagic layer. At the entrance of the Bay of Villefranche (NW Mediterranean Sea), Weinbauer et al. [27] examined TEP seasonal variability at a depth of 300 m. They found that the TEP volume concentration varied within a range of 0.1–0.6 ppm, which increased with temperature.

### 2.3. Summary of the Observed Data

- In the mesopelagic layer, the variability range of the TEP concentrations is on the order of 100-fold (Table 1). In the marginal sea and slope region, a vertical (depth-dependent decrease [18,19]) and a lateral (offshoreward decrease [17]) gradient in the TEP distribution pattern has been documented. Microscopic observations have revealed that TEP are colonized by prokaryotes in the mesopelagic layer [17,24].
- Examination of the full-depth distribution of TEP in open oceans has revealed that TEP concentrations are less variable (<3 fold) throughout the meso- and bathypelagic water columns down to the depths of 4000–5400 m (Table 1), although some anomalous features have been noted [22,25]. This vertical distribution of TEP is largely decoupled from the distribution of prokaryote abundance and production [19] (Figure 1). One study using the microscopic method has found a remarkably high TEP abundance in the bathypelagic layer of the coastal upwelling region [25]. Microscopic observations have also found that TEP were colonized by prokaryotes in the bathypelagic layer. High relative contributions of TEP-associated prokaryotes to the total prokaryote abundance (up to 20%) were observed at the depths >2000 m in the Arctic Ocean [24].

## 3. Potential Factors Affecting TEP Distribution in the Deep Oceans

As a basis for examining possible mechanisms underlying the observed TEP distribution, here we discuss sources (transport and autochthonous production) and sinks (microbial degradation and grazing) of TEP in deep waters.

### 3.1. Sources of TEP

#### 3.1.1. Transport

Individual TEP generally sink only slowly, or even float, due to their low density and high porosity [5]. However, if they are incorporated into or associated with dense sinking particles, TEP can be transferred to the deeper layers by gravitational settling. TEP are likely to be released (disaggregated) from sinking particles during their transit in the meso- and bathypelagic water columns. The hydrolysis of the TEP and other polymer networks by ectoenzymes produced by colonizing prokaryotes may enhance the fragmentation of the sinking particles [28]; however, physical processes (turbulence and sheer) and the disturbance caused by zooplankton may also promote fragmentation [26,29].

In oceans, the vertical POC flux (F, mg C m^−2^ d^−1^) attenuates with depth (Z m). The most common model describing this attenuation is the power law: F = (F_100_/100) × Z^−0.858^, where F_100_ is the POC flux at a depth of 100 m [30]. The equation indicates that, of the POC removed from the upper layer, 14% reach a depth of 1000 m, and only 4% are found at a depth of 5000 m. This substantial attenuation of the POC flux with depth is a consequence of fragmentation and remineralization of POC during the transit of particles through the water column [30,31]. Although no previous work has examined the vertical flux attenuation of TEP, the data obtained by Passow et al. [32] support the notion that the depth-dependent attenuation of TEP flux is high. Using time-series sediment traps deployed at a depth of 500 m in the Santa Barbara Channel, they demonstrated that the daily recovery of TEP from sediment traps relative to the TEP standing stocks in the upper water column (0–75 m) was less than 2% (mostly, <0.5%). These values were comparable to those of POC. Therefore, the extent of vertical delivery of TEP associated with settling particles appears to decrease rapidly with depth.

As already mentioned, Yamada et al. [19] found that the TEP concentration decreased with depth in the Arctic slope region. Similarly in the Mediterranean Sea, TEP concentration tended to decrease with depth in the meso- and bathypelagic layers [18]. These depth-dependent trends were interpreted as an indication that the TEP distribution is shaped by the vertical delivery mediated by sinking particles. However, in the open ocean domains of the Pacific and Atlantic Oceans, the depth-dependent variability in TEP concentration was small [19,22] (Figure 1). These apparently contradictory results suggest that the coupling of TEP vertical distribution and sinking POC flux attenuation differs, depending on the environment, such that it is strong in marginal seas and slope regions but weak in open oceans. One could then hypothesize that the vertical delivery of TEP is enhanced by ballast particles (e.g., dense mineral grains) supplied from land and continental shelves and slopes, leading to a magnified effect of sinking particles on TEP vertical distributions in marginal seas and slope regions.

Particle settlement is not the sole physical mechanism by which POC (including TEP) is delivered to deeper waters. Non-sinking particles can be delivered to deeper layers via mixing, convection, and lateral transport along the slope of isopycnal surfaces [33]. The TEP distribution at a particular depth may also be influenced by the transport driven by intermediate water intrusions and deep water currents. In this regard, the anomalously higher TEP concentration and abundance found in the bathypelagic layer relative to the mesopelagic layer is intriguing [22,25]. We clearly need more data concerning the variability in TEP concentrations across different water masses in the meso- and bathypelagic oceans.

#### 3.1.2. Autochthonous Production of TEP

Potential TEP producers in deep oceans are bacteria and archaea (prokaryotes) [6]. Based on the results from a study conducted in the Mediterranean Sea, Ortega-Retuerta et al. [18] suggested that prokaryotes release TEP during their growth. They examined the changes of TEP concentrations in seawater cultures (filtered seawater amended with in-situ prokaryote communities) prepared using meso- and bathypelagic waters. During the 6-day incubation period, they found that the TEP concentration increased with prokaryote abundance. Additionally, the TEP concentration was positively correlated with prokaryote abundance in the meso- and bathypelagic layers. Based on the data, the authors suggested that prokaryotes played a role as a source of TEP in the deep Mediterranean Sea.

The self-assembly of TEP precursors in seawater is another potential autochthonous mechanism of TEP production. Gels are thought to be produced by the spontaneous assembly of polymers to form nanogels in seawater and may contribute to the DOC-to-POC transition [2,3,4]. Nanogels become larger due to annealing and subsequent coagulation [4,26]. Because high-molecular-weight DOC, containing polymeric substances, is distributed throughout the deep water column [34], the self-assembly of polymeric precursors may explain the uniform TEP vertical distribution observed in the deep waters of the Pacific [19] and Atlantic Oceans [22]. In support of this hypothesis, Ding et al. [35] found that gels were formed by polymer self-assembly in deeper waters of the subtropical Pacific. The concentration of self-assembled gel was similar at depths of 500 and 4000 m. However, currently, there is no evidence that the gels detected by the Ca^2+^ binding assay used by Ding et al. [35] were TEP. According to the gel theory, counteriron interactions take place between Ca^2+^ and marine biopolymers, leading to gel production in the ocean [3]. To resolve a critical question concerning whether this theory explains TEP production in deep oceans, further studies are required to eliminate inherent ambiguities of the results obtained by the Alcian Blue assay.

### 3.2. Sinks of TEP

#### 3.2.1. Prokaryotes

In the previous section, we discussed the possibility that prokaryotes produce TEP. However, prokaryotes may also act as decomposers of TEP, whereby they are regarded as a sink of TEP. Several studies have demonstrated that prokaryotes in the meso- and bathypelagic waters express a wide range of ectoenzymatic activities (including phosphatase, beta-glucosidase, and peptidases) that cleave polymeric chains [13]. It is also generally known that the genes coding prokaryotic enzymes that cleave sulfate residues (sulfatase) and carboxyl residues (carboxylase) are widespread and expressed in marine microbes [36]. However, a recent study suggested that a fucose-containing sulphated polysaccharide excreted by diatoms is less susceptible to enzymatic degradation relative to non-sulphated polysaccharide [37]. Therefore, a question arises regarding the lability of TEP in deep waters: Are they good substrates for prokaryotes or not?

Bulk DOC in seawater, especially in deep water, turns over slowly, with the average lifetime being on the order of millennia [1,38]. Although the mechanisms controlling the persistence of DOC have been a subject of much debate, one theory suggests that the inherently recalcitrant molecular properties of DOC explain its resistance to microbial attack [39,40]. Thus, if TEP in deep waters are primarily formed via the spontaneous assembly of DOC [2,4], one would expect that TEP in deep waters are recalcitrant (unavailable for prokaryote consumption). Alternatively, if TEP are formed from or enriched by labile constituents presumably derived from sinking particles [41], TEP may act as “hot spots” for prokaryote substrate consumption. Although we have no conclusive answer regarding this question, the observations of Bar-Zeev et al. [17] and Busch et al. [24] that TEP at depth were colonized by prokaryotes appear to support the “hot spot” hypothesis. Future studies examining the community compositions and gene expression of prokaryotes associated with TEP in deep water may provide insight into this topic. However, for rigorous testing of this hypothesis, we clearly need data about the metabolic activities of the TEP-associated prokaryotes. Currently, no standard method is available to determine the TEP consumption rate by microbes in seawater, making it difficult to evaluate quantitatively the role of prokaryotes as a sink of TEP. Efforts to determine TEP consumption by deep water prokaryotes are further complicated by challenges in evaluating the effects of high hydrostatic pressure on microbial physiology [42,43].

#### 3.2.2. Grazers

Very little is known about the grazing of TEP by metazoan and protist grazers in deep waters. Given that TEP in the meso- and bathypelagic layers are colonized by prokaryotes and that prokaryotes are protein-rich constituents with a low carbon-to-nitrogen ratio [44], the TEP–prokaryotes complex may serve as a good food source for detritivorous grazers. The search for TEP–prokaryotes complexes by metazoan grazers may be facilitated by bacterial bioluminescence [45]. The possibility of the food web control of TEP in the dark ocean deserves further investigation.

## 4. Organic Carbon Inventory

Yamada et al. [19] estimated the organic carbon concentration of TEP (TEP-C concentration) in the meso- and bathypelagic waters. They found that TEP-C represents a substantial fraction of the POC inventory. In the mesopelagic layer, TEP-C concentrations were 10–23 μg C L^−1^, accounting for 230% and 320% of the POC in the Arctic and Pacific Oceans, respectively. The corresponding values in the bathypelagic layer of the Pacific Ocean were 41 μg C L^−1^ and 550%. Similarly high-to-moderate contributions of TEP-C to POC in the meso- and bathypelagic layers have been noted in other studies [17,18,21]. Hypotheses to explain the fact that the concentration of TEP-C exceeds POC are listed below.

1. To estimate TEP-C, studies have used a conversion factor derived from laboratory experiments using diatom cultures [15]. However, the validity of this conversion factor in deep waters has yet to be tested. If the organic carbon yield relative to the Alcian blue-reactive residues (sulfate and carboxyl groups) of TEP is systematically lower in deeper than shallower waters, the TEP-C values estimated from the conversion factor for the shallower water (diatom-derived fresh TEP) may be too high.
2. TEP-C concentration may exceed POC concentration due to the use of different pore-size-filters for the determination of TEP (0.4-μm-pore-size polycarbonate filter) and POC (0.7-μm-pore-size GF/F filter). If large quantities of organic carbon associated with TEP pass through the GF/F filters, but are retained on 0.4-μm polycarbonate filters, this would explain the high TEP-C concentration relative to POC.

Engel et al. [25] estimated the TEP-C concentration using the microscopic data and an equation that relates the size (ESD) of TEP to the organic carbon concentration [44]. TEP-C in the meso- and bathypelagic layers at the six stations examined were in the range of 0.6–1.7 μg C L^−1^ and 0.4–3 μg C L^−1^, respectively, except for higher values found in the Mauritanian upwelling region where the corresponding values were 48 and 22 μg C L^−1^, respectively. These TEP-C values, except for those from the Mauritanian upwelling, were about one order of magnitude lower than those reported by Yamada et al. [19]. The discrepancies between the two studies may be explained by regional and seasonal differences, uncertainties associated with the TEP-to-carbon conversion factor or equation, and the difference in the lower size limit of the TEP determination, which was 0.4 μm in Yamada et al. [19] and 1 μm in Engel et al. [25].

**Figure 2:**
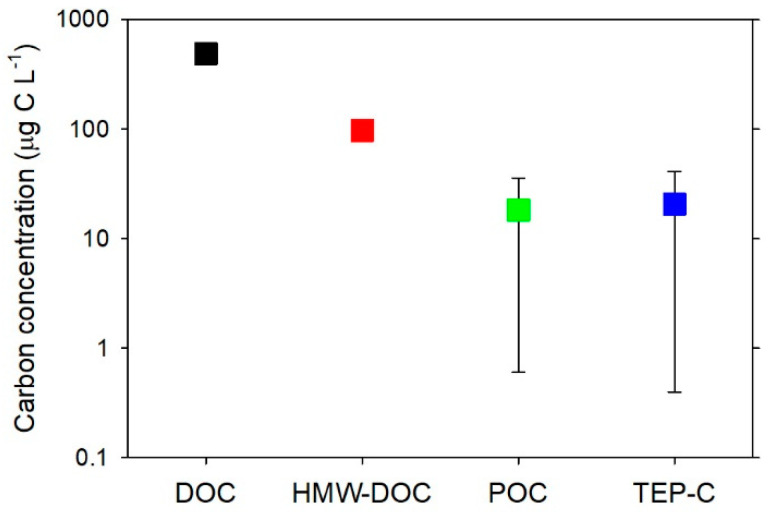
Concentration ranges of DOC, high-molecular-weight DOC (HMW-DOC), POC, and TEP-C in the bathypelagic layer. Error bars represent the minimum and maximum values reported in the literature and the symbols indicate the midrange values. DOC and POC values are from Nagata et al. [13]. TEP-C is from Yamada et al. [19] and Engel et al. [25]. The large ranges for POC and TEP-C reflect both regional and seasonal variabilities. In addition, there are methodological uncertainties in the POC determination (e.g., [46]) and TEP-C estimation. Although analytical errors associated with colorimetric and microscopic methods of TEP estimation are generally small (standard deviations of replicated measurements are typically <10% of the mean values [8,15,19]), there are uncertainties in the conversion factor (or equation) relating TEP to carbon (see text). The range of DOC is smaller than the size of the symbol. HMW-DOC was assumed to be 20% of DOC [34].

Figure 2 compares TEP-C concentrations with other organic carbon pools in the bathypelagic layer. Broadly, TEP-C accounts for 0.1–3% of DOC, 1–10% of high-molecular-weight DOC, and 10–>100% of the POC. Although we still have much to learn about the organic carbon stock associated with TEP, the available data suggest that TEP-C is a significant organic carbon pool in deep waters.

## 5. Knowledge Gaps and Future Challenges

**Figure 3:**
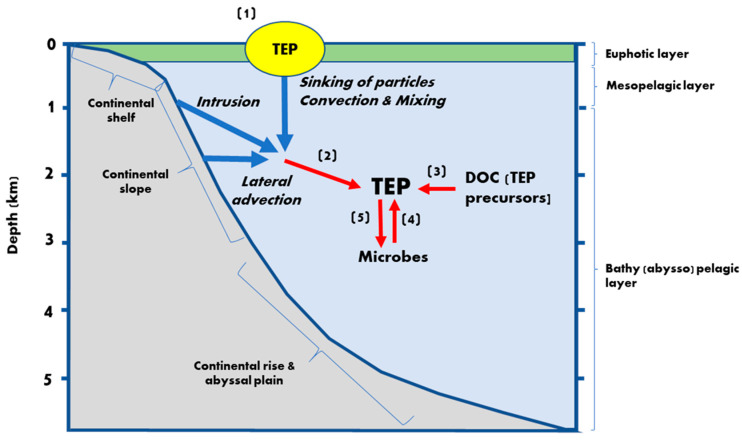
Schematic representation of the major processes involved in the TEP dynamics in the deep ocean. (1) TEP are mainly produced by phytoplankton and bacteria in the upper ocean [5,6]. (2) A fraction of TEP produced in the upper ocean is transported to the deeper layers. The transport processes include the sinking of particles and the advective transport due to convection and mixing, and intrusion and lateral advection. TEP transport mediated by these processes is more important in the ocean’s margins than in open ocean domains. (3) TEP may be produced by the coagulation of nanogels, which are formed via the spontaneous assembly of DOC [2,3,4]. The ultimate source of DOC is primary production in the upper ocean, yet chemical characteristics of DOC and mechanisms underlying the persistence of DOC over millennia are poorly understood [1,38]. (4) Microbes may produce TEP, (5) while they also consume TEP. TEP are colonized by prokaryotes, acting as “hot spots” of microbes in deep oceans.

Figure 3 summarizes the major processes involved in the TEP dynamics in the deep oceans. There are large knowledge gaps to be filled if we are to fully understand the role of TEP in the carbon cycles in meso- and bathypelagic oceans. The available data on TEP distribution at depth are scarce, especially in open oceans, hampering coherent examination of the relationship between water mass structure and TEP abundance. Data are also limited regarding the vertical and lateral transport of TEP, which severely limits our understanding of TEP dynamics in the oceans. Although the detailed, extensive examination of TEP in meso- and bathypelagic realms is a challenging task, the collected data should provide clues to evaluate the sources and sinks of TEP. Here, we list some research areas that deserve further investigation.

1. Theories have been proposed to explain the spontaneous assembly of gels [2,3,4] and the coagulation of particles [26] in seawater. Self-assembled gels have been identified in the deep oceanic water column [35]; however, a rigorous validation of TEP quantification methods are required to evaluate TEP formation via the spontaneous assembly of DOC. Coagulation theory is generally formulated to describe the coagulation rate as a product of particle number, collision rate, and stickiness, whereby the dominant mechanism by which the collision rate is controlled differs, depending on the particle size. Data on TEP size distributions in deep oceans are scarce [25], and we lack information about the abundance of TEP or TEP precursors in the sub-micrometer size range. Previous work has revealed that submicron particles and colloids are present in meso- and bathypelagic oceans [47,48,49], yet it remains to be seen if TEP are produced via the coagulation of submicron particles under deep water physical conditions. Disaggregation, the converse process of aggregation, may also affect TEP distribution at certain depths. Further studies are required to evaluate the extent of TEP delivery via the disaggregation of sinking particles.
2. To date, only a few studies have used the microscopic method to examine prokaryote colonization on TEP at depth [17,24]. These studies have provided valuable information regarding the potential role of TEP in the food webs of deep waters. Given that deep water microbial communities are dominated by organisms with surface-associated lifestyles, as evidenced by the presence of genes encoding pilus, polysaccharide, and antibiotics synthesis [36], it is likely that TEP in deep waters represent a hot spot of microbes, including prokaryotes, protists, and viruses [13]. They can also serve as important food resources for metazoan grazers that thrive throughout the oceanic water columns [50]. Despite the extensive data collected over the past two decades concerning prokaryote, protist, and virus distributions in deep water columns [12,13], further research is needed to incorporate TEP and other gel-like particles into the food web models of deep oceans.
3. To incorporate TEP dynamics into ocean carbon cycle models, it is necessary to collect quantitative data on TEP in terms of carbon. In this regard, further testing and refinement of methodologies are required to reduce large uncertainties associated with the estimation of TEP-C. It is also important to clarify the mechanisms by which TEP dynamics are regulated and to evaluate the turnover time of TEP. Currently, TEP turnover time and their lability in deep waters is poorly understood, suggesting a need to develop new methods to tackle this issue. Efforts to determine the dynamics (production and decay) of detrital polysaccharides in marine waters are inherently complicated by numerous analytical challenges [51]. We clearly need to know more about the chemical compositions, physical structures, and microbial processing of TEP and other gels in deep waters.

To conclude, major future challenges include the improvement of our understanding of the ocean carbon fluxes mediated by TEP at depth. The applications of gel theory and coagulation models under deep water settings are probably most effective when they are assisted by an enhanced understanding of the quantities, transport, turnover, and chemical properties of TEP. Furthermore, better recognition and determination of microbial processing of TEP are necessary for a complete understanding of TEP-mediated biogeochemical and ecological processes in deep oceans.

## Figures and Tables

**Table 1:** TEP concentrations (μg Xeq. L^−1^) in the meso- and bathypelagic layers. Values in parentheses are the depth range in meters.

Region	Mesopelagic		Bathypelagic		References
Coastal and slope region, estuary and marginal sea					
Santa Barbara Chanell (eastern Pacific)	20	(200–1400)			[15]
Eastern Mediterranean Sea	200	(300–1000)			[17]
Mediterranean Sea and Atlantic	1.2–35	(200–1000)	0.6–16	(1000–3900)	[18]
Western Arctic (slope region) ^1^	37–129	(200–1000)	39–52	(1230–1960)	[19]
St Lawrence Estuary	15–200	(130–320)			[21]
Open oceans					
North Atlantic Ocean (subtropical) ^2^	18–33	(200–1000)	16–48	(1250–4580)	[22]
Central Pacific (subtropical and equatorial) ^1^	12–40	(200–1000)	14–34	(1000–5370)	[19]

^1^ Values from the original data of Yamada et al. [19]; ^2^ Values extracted from Figure 13 of Cisternas-Novoa et al. [22].

## Data Availability

Not applicable.
